# Primary Adrenal Leiomyosarcoma: a Case Report and Literature Review

**DOI:** 10.4137/cmo.s627

**Published:** 2008-04-28

**Authors:** Mencoboni M, Bergaglio M, Truini M, Varaldo M

**Affiliations:** 1 Medical Oncology Unit, “Villa Scassi” Hospital, Genova, Italy; 2 Medical Oncology Unit, “Villa Scassi” Hospital, Genova, Italy; 3 Pathology Unit, “National Cancer Institute”, Genova, Italy; 4 Urology Unit, “Villa Scassi” Hospital, Genova, Italy

## Abstract

The case presented here illustrates a 75 year old female patient who underwent surgical resection of a right adrenal mass of uncertain nature. The final histological diagnosis was consistent with leiomyosarcoma arising from the adrenal anatomic site.

Primary leiomyosarcoma of the adrenal gland is a very rare malignant mesenchymal neoplasm: to our knowledge, this is only the twelfth case reported in literature.

We describe the clinical course and a brief review of clinical and histological features, biologic behaviour, diagnostic approaches and therapeutic strategies.

## Case Report

A 75-year-old white female was admitted to the Department of Urology of our hospital following the ultrasound finding of a right adrenal mass of uncertain nature. Her medical history was unremarkable, except for long-term essential arterial hypertension. For a month prior to hospitalization the patient had complained of persistent and progressive epigastric and right loin pain. Physical examination showed only mild abdominal discomfort at palpation. No enlarged lymph nodes were present. Laboratory tests detected no abnormalities. Urinary levels of vanilmandelic acid, catecolamines and plasmatic level of aldosterone were normal.

**Figure 1:**
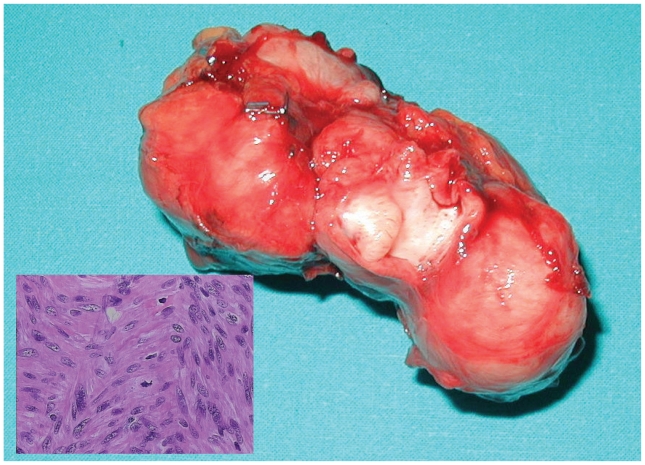
Gross appearance of the roundish, fibrous neoplasia. Insert: neoplasia with fascicular growth pattern (E.E. 40×).

Chest radiograph and electrocardiogram revealed no pathological findings. Upper gastrointestinal endoscopy detected oesophageal hiatal hernia with reflux oesophagitis, and total-body CT confirmed the presence of a right adrenal mass of about 5 × 4 cm, suspected to be an adrenal carcinoma, so radical surgery was planned and performed with the resection of a roundish formation from the adrenal anatomic site. Normal adrenal gland was not visible (Fig. 1).

Three days after surgery the patient developed a pulmonary embolism: an inferior vena cava thrombosis was identified by angio-CT scan. Pulmonary embolism was identified by angio-CT scan, too. Moreover, an unexplained progressive drop of hemoglobin concentration down to 7.7 g/dl, requiring infusion of two units of packed red cells, was observed. The patient was transferred to our Intensive Care Unit, where she received routine treatment for pulmonary embolism including anticoagulant therapy first with intravenous heparin and then with warfarin.

The clinical course was satisfactory, and subsequent Doppler-ultrasonography confirmed the return to normal of venous flow through inferior vena cava. The patient, clinically stable with a good cardio-respiratory compensation, was discharged on postoperative day 18; continuation of anticoagulant therapy and physical rehabilitation were recommended. No oncological treatment was provided after surgery, and no evidence of recurrent disease was observed one year later.

Gross pathological examination of the resected tissue showed a roundish, fibrous neoformation of 8 × 4 × 5 cm. A thrombus was detected inside the adrenal vein; this thrombus was not visible on CT performed before surgery. On sectioning of the mass, no adrenal structure was recognizable. Resection margins were free of disease. Histologically, tissue sections showed a neoplasm consisting of elongated cells with eosinophilic cytoplasma and elongated nuclei with rounded ends, small nucleolus with moderate polymorphism; the cells are arranged in fascicles; on average, 16/50 HPF mitoses were observed. No necrotic areas were detected (Fig. 1).

**Figure 2:**
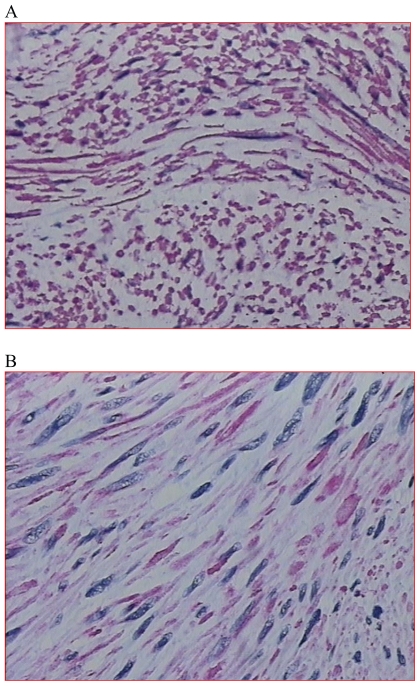
(**A**) Positive Desmin immunostaining (20x); (**B**) Positive Muscle Specific Actin immunostaining (40×).

Neoplastic cells were strongly and diffusely positive immunohistochemical staining for desmin, SMA(1A4)(-Smooth Muscle Actin (1A4) Rimary Antibody) and actin (HHF3 Muscle-Specific Actin -HHF35- Primary Antibody 5-) (Fig. 2); immunohistochemical staining for CD34 and CD117 was negative. Ki-67 nuclear antigen (monoclonal MIB1 Antibody) was 20%. The final histological diagnosis was consistent with leiomyosarcoma (UICC: pT2b/G2) arising from the adrenal anatomic site and substituting adrenal gland. An adrenal origin was thus deemed very likely.^1^,^2^

Intra-abdominal soft-tissue tumours represent only 0,1%–0,2% of all adult malignancies; among them leiomyosarcoma is the most common histological subtype, accounting for approximately 25%–30% of cases. However, primary leiomyosarcoma of the adrenal gland is a very rare malignant mesenchymal tumour: to our knowledge, this is only the twelwfth case reported in literature.^3^,^4^,^5^,^6^,^7^,^8^,^9^,^10^,^11^,^12^

The aetiology of these neoplasms is unclear. A growing incidence of smooth muscle tumours in HIV and Epstein-Barr virus positive patients has been reported, but nothing certain is known about the pathogenetic involvement of these viruses.^13^ Despite the slow growth and late metastasis, leiomyosarcoma still carries a poor prognosis. The tumour may grow to a large size before directly invading adjacent structures; systemic spread occurs late, but there is a high incidence of local recurrence.

At present, the prognosis for leiomyosarcoma patients is not predictable. Survival depends on tumour size, location, feasibility of complete resection^14^ and morphologic grading (presence of mitotic activity, necrotic areas, nuclear atypia).^15^,^16^ The morphology-based grading system alone makes it difficult to predict outcome, although in some studies tumour size and histological grade have correlated with biologic behaviour. In fact, little is known about the mechanisms underlying the development of aggressive tumour behaviour, and about the molecular genetic changes associated with clinical outcome. Chromosomal aberrations may contribute to morphological changes in soft-tissue leiomyosarcomas,^17^,^18^ as these alterations of gene expression appear to be correlated with the tumour’s clinical behaviour and histological grade. The striking dissimilarities in the gene expression patterns of leiomyosarcomas of varying differentiation status and clinical aggressiveness seem to suggest that several and different genetic abnormalities are responsible for the genesis and progression of this tumour.^19^ For instance, 13q14-q21 loss and 5p14-pter gain at diagnosis could be used to identify patients with leiomyosarcoma who are likely to have a shorter survival and who might benefit from early treatment intensification. Analysis of the RB-1 genes and proteins in the Rb-cyclinD pathway has revealed frequent abnormalities in leiomyosarcomas.

Because of the prolonged time interval separating the diagnosis of the primary tumour and the involvement of distant sites, an accurate and early diagnosis of these neoplasms must be made. Indeed, improved diagnostic approaches are enabling the early diagnosis and surgical treatment of an increasing number of cases. Radical surgical resection remains the only proven therapeutic modality that prolongs the survival in patients with leiomyosarcoma.^15^,^20^ Histological grade often correlates with biological behaviour and with prognosis. The principal sites of metastasis are lungs and liver; regional lymph node metastasis are rare.

Postoperative adjuvant radiation therapy is recommended for the treatment of locally advanced malignancy.^21^ The efficacy of chemotherapy is poorly defined and very limited; pre-operatively it can be performed in cases of inoperable tumours, whereas in the post-operative setting it may be suggested for incompletely resected or metastatic tumours. In patients with locally advanced or metastatic disease any one of the many schemes containing an anthracycline and/or ifosfamide may be considered the standard.^15^ Few chemotherapeutic agents are active as second-line treatment. Gemcitabine, taxans and others are considered quite ineffective, although responses to these agents have been reported in rare cases.^22^,^23^

Conclusively, complete surgical resection followed by a combination of radiotherapy and chemotherapy may constitute the optimal treatment for advanced leiomyosarcoma to ensure long-term survival, even if recurrences are frequent.^15^,^24^ In our case, the tumour had completely replaced the right adrenal gland but no other structure appeared to be infiltrated.

Treatment was therefore limited to surgical resection with no oncological treatment provided after surgery, and no evidence of recurrent disease was observed one year later.
